# Prostate Cancer-Associated Fibroblasts: A Review on CAF Functions, Heterogeneity, Resistance Mechanisms, and Future in a Chip

**DOI:** 10.3390/ijms27031585

**Published:** 2026-02-05

**Authors:** Nikolett Lupsa, Erika Heninger, Adeline B. Ding, Cristina Sanchez De Diego, Katherine Vietor, Shannon R. Reese, Aaron M. LeBeau, David Kosoff, David J. Beebe, Sheena C. Kerr, Joshua M. Lang

**Affiliations:** 1 Department of Medicine, School of Medicine and Public Health, University of Wisconsin, Madison, WI 53705, USA; 2 Carbone Cancer Center, School of Medicine and Public Health, University of Wisconsin, Madison, WI 53705, USA; 3 Cancer Biology Graduate Program, McArdle Laboratory for Cancer Research, School of Medicine and Public Health, University of Wisconsin, Madison, WI 53705, USA; 4 Department of Pathology & Laboratory Medicine, School of Medicine and Public Health, University of Wisconsin, Madison, WI 53705, USA; 5 William S. Middleton Memorial Veterans Hospital, United States Department of Veterans Affairs, Madison, WI 53705, USA; 6 Department of Biomedical Engineering, College of Engineering, University of Wisconsin, Madison, WI 53706, USA

**Keywords:** prostate cancer, cancer associated fibroblasts, tumor microenvironment, CAF heterogeneity, therapy resistance, organ-on-a-chip models

## Abstract

Cancer-associated fibroblasts (CAFs) are key regulators of the prostate tumor microenvironment (TME) with influence on disease progression and therapeutic response. CAFs originate from multiple precursors and retain remarkable plasticity while tumors evolve. Therefore, the CAF pool displays considerable functional heterogeneity, which is well-reflected in complex molecular signatures. However, overlapping biomarker patterns with other stromal subsets make it challenging to identify and assess the role of specific CAF subpopulations. Through reciprocal tumor–stroma interactions, CAFs promote extracellular matrix (ECM) remodeling, angiogenesis, metabolic reprogramming, and immune evasion, collectively fostering an adaptive niche that supports tumor survival, though some CAF subsets have been shown to support anti-tumor response. In prostate cancer (PCa), CAFs promote resistance to androgen receptor pathway inhibitor therapy, chemotherapy, and radiotherapy, emphasizing their potential value as therapeutic targets. However, CAF targeting has shown limited clinical benefit in PCa, due to complex, context-dependent CAF functions that make it challenging to exploit this unique stromal population for therapeutic gain. Recent advances in organ-on-a-chip (OOC) models offer new opportunities to investigate the mechanisms behind TME interactions and evaluate CAF-targeted strategies in physiologically relevant fully humanized environments. This review provides current insights into CAF heterogeneity and therapy resistance in PCa and highlights emerging translational OOC models to guide the development of more effective therapies to disrupt the TME.

## 1. Introduction

Globally, despite a decline in mortality rates attributed to Prostate Specific Antigen (PSA) screening, prostate cancer (PCa) remained the second most commonly diagnosed malignancy and the fifth leading cause of cancer-related death in men in 2022 with 397,000 deaths and an estimated 1.5 million new cases [1]. Examining age-standardized (incidence and mortality) rates (ASRs), PCa has the second highest ASR in men in high Human Development Index (HDI) countries, behind lung cancer, and the first highest ASR in men in low HDI countries [1]. PCa is a hormone-driven disease. Therefore, progression heavily relies on androgen receptor signaling, and hormone therapies such as androgen deprivation therapy (ADT) and androgen receptor pathway inhibitors (ARPI) are first-line options in standard of care treatments with remarkable benefit. However, treatment resistance is inevitable and a considerable cohort of patients on hormone therapies develop castration-resistant prostate cancer (CRPC) and require additional treatments, including chemotherapy, radiotherapy, or targeted therapies [2,3]. Aggressive forms of PCa have been linked to intrinsic features of the tumor, such as genomic mutations in *BRCA2*, *TP53*, *PTEN*, and *RB1*, among other driver genes [4] and androgen receptor (AR) splice variants that lead to genomic instability and contribute to treatment resistance [5,6,7]. Additionally, there is growing evidence demonstrating significant contributions of the tumor microenvironment (TME) that influence PCa tumor progression and therapeutic response. Tumor-promoting subsets of the surrounding stroma, including suppressive immune cells and cancer-associated fibroblasts (CAFs), have been shown to be enriched in tumors that associate with poor clinical prognosis, which include progression to CRPC, treatment resistance, and high-grade metastatic disease [8]. Despite the availability of numerous FDA-approved treatments, advanced metastatic CRPC remains incurable. This highlights the need for novel biomarkers and more effective therapeutic strategies that include disruption of tumor-promoting TME networks and targeting TME-driven treatment resistance mechanisms.

In recent years, the PCa TME has been extensively studied to better understand its role in driving tumor progression, immune evasion, and treatment resistance, though the TME has also been associated with anti-tumor activity [9,10]. CAFs perform multiple functions that promote tumor survival and induce dynamic alterations in the TME networks and metabolism that are linked to therapeutic response patterns. Therefore, CAFs have been assessed as enticing targets to enhance therapeutic efficacy and reverse treatment resistance in PCa [11]. CAFs are known to remodel tissue architecture, create new physical barriers, and limit anti-tumor immune responses. However, as a testament to the dynamic nature of CAFs, there is also evidence that supports anti-tumor CAF functions in solid tumors including PCa [9,10,12,13]. For example, studies in pre-clinical models of pancreatic adenocarcinoma (PDAC) demonstrated that CAF depletion contributed to disease progression [14,15]. These versatile functions create an adaptable multicellular niche around tumors with a unique spatial organization that evolves with tumor progression. However, research into the role of CAFs in PCa pathogenesis is limited by the lack of translationally relevant TME preclinical models that can accurately reflect the complex interactions and site-specific heterogeneity found in the context of the native tumor architecture in the prostate gland or at distant metastatic sites.

In this review, we focus on recent advances in the identification and characterization of CAFs. Furthermore, we provide an overview of function to gain a deeper understanding of the role of CAFs in PCa tumorigenesis. Finally, we discuss recently developed in vitro, organ-on-a-chip model systems designed to investigate mechanisms by which CAFs contribute to drug resistance.

## 2. Normal Prostate Fibroblasts

The prostate architecture is made of ducts and acini nestled within fibromuscular stroma [16]. The glands are lined with columnar luminal cells over basal cells that sit atop a basal lamina, which is an essential barrier between the epithelium and the surrounding stroma [17,18,19]. The basal lamina plays a critical role in maintaining tissue integrity, resisting tissue damage and invasion. Beneath the lamina is a diverse subset of stromal cells, such as fibroblasts, smooth muscle cells, endothelial cells, pericytes, and immune cells [18,20].

Prostate development is dependent on androgens, and the activation of AR in the urogenital sinus mesenchyme is essential for prostatic epithelial proliferation and differentiation [21]. Following induction by the epithelial cells, prostate stroma mainly differentiates from smooth muscle cells, resulting in heterogenous populations of smooth muscle cells, mesenchymal cells, or fibroblasts [22,23]. The dynamic interplay between fibroblasts and epithelial cells is essential for both early differentiation and organ development, as well as for maintaining normal prostate function throughout adulthood [24]. This includes secretion of an elastic, collagen-based ECM to facilitate adaptation to tissue injury and tissue repair [25,26]. Additionally, prostate fibroblasts secrete growth factors and signaling molecules that regulate tissue remodeling, tissue homeostasis, tissue metabolism, and ECM polarization [26,27]. Furthermore, fibroblasts in solid tissues are critical for the recruitment and polarization of immune cells, regulation of cellular interactions in a tissue localization-specific manner and contribute to signaling in stem cell niches [28]. However, normal prostate fibroblasts are not a uniform cell subset and express several proteins, including vimentin, CD90, S100A4 and others, also produced by many other cell types of different tissue origins [23]. Using single-cell RNA sequencing (scRNA-seq), Joseph et al. described two main phenotypic subcategories of normal prostate fibroblasts: “peri-epithelial” or *APOD^+^* and “interstitial” or *C7^+^*. “Peri-epithelial” or *APOD^+^* fibroblasts are linked to marked expression of *APOD*, *PTGDS*, *PTGS2* and *MMP2* driver genes, while “interstitial” or *C7^+^* fibroblasts dominantly express *C7*, *CCK*, *PCOLCE2* and *GSN*. Furthermore, tissue biomarker analysis linked the spatial distribution of these distinct subsets to their respective functional roles [29]. Peri-epithelial fibroblasts encase glands and create a local WNT_low_ niche, while interstitial fibroblasts reside in ECM-rich zones and emphasize matrix programs and expression of growth factors like *IGF1* and *FGF2*. In the healthy prostate, stromal fibroblasts reside in a low-proliferative, homeostatic state within the fibromuscular stroma that supports epithelial structure and ECM turnover [23]. When epithelial injury or inflammation occurs, local prostate fibroblasts activate into αSMA^+^ (alpha smooth muscle actin) myofibroblasts and engage a wound-repair program that closely resembles “granulation tissue”, a type of connective tissue that forms on the surface of healing wounds. TGFβ1 is a key driver of this transition in human prostate fibroblasts [30]. After acute injury, this reactive stromal response can regress. However, chronic or recurrent inflammation in the prostate has been associated with sustained fibroblast activation, leading to excess ECM deposition and fibrosis that disrupts tissue architecture and function [31,32]. These ECM changes subsequently result in the loss of other tissue-resident cells, such as well-differentiated epithelial and smooth-muscle cells, and lead to organ dysfunction. 

## 3. Cancer-Associated Fibroblasts

### 3.1. CAF Origins

Normal prostate stroma provides physical structure and barriers that control epithelial proliferation and invasion during early tumorigenesis [33]. However, during the early transition to PCa, tumor cells hijack the regulatory process of tissue repair, displace normal stroma and perpetuate “endless” wound healing processes that enable the exploitation of cellular resources and promote tumor growth [20]. Tumor cells also induce molecular changes in the stroma that replace smooth muscle cells with activated fibroblasts and myofibroblasts, which form a distinct group of tumor-associated fibroblasts also referred to as CAFs [30]. During this stromal reprogramming, the continued loss of differentiated smooth muscle cells ultimately elevates CAFs to become the most abundant cell type in the prostate TME [34]. Although CAFs share functional similarities with normal fibroblasts, CAFs uniquely promote the formation of reactive stroma, which enhances tumor-induced alterations in ECM deposition, neovascularization, and immune infiltration. Although CAFs are mainly derived from the mesenchyme, they display lineage plasticity and may also originate from [25] endothelial cells, pericytes, and bone marrow-derived mesenchymal stem cells (MSCs), which further contributes to the CAF heterogeneity captured in variable and complex biomarker signatures.

PCa-driven differentiation of MSCs into CAFs has also been studied in 3D models systems. PCa-derived exosomes were shown to inhibit classical adipogenic differentiation of bone marrow MSCs while promoting an α-SMA^+^ myofibroblast/CAF-like phenotype, characterized by elevated secretion of VEGF-A, HGF, and MMP-1/-3/-13 ECM remodeling factors. These CAFs were pro-angiogenic and enhanced tumor cell proliferation and invasiveness [35].

The crosstalk between CAF and tumor cells is partly regulated by Yes-associated protein 1 (YAP1), which forms a complex with TEAD1 to promote SRC proto-oncogene expression by influencing actin–cytoskeletal proteins such as cortactin, focal adhesion kinase, and paxillin. YAP1 is associated with normal prostate fibroblast-to-CAF conversion, enhancement of tumor growth, and metastasis. Reduction in YAP1 expression decreases SRC phosphorylation which leads to diminishing SRC activity. Therefore, silencing YAP1 has been identified as a potential targetable approach to inhibit normal fibroblast-to-CAF conversion and prostate tumor growth [36].

Differential cytoskeletal structures in CAFs have been associated with increased F-actin fiber alignment and stiffness as compared to prostate fibroblasts in normal adjacent tissue [37]. Additionally, in a co-culture setting, interactions with CAFs were shown to promote cytoskeletal adaptations, an elongated morphology, invasiveness, and proliferation in non-tumor epithelial cells. Therefore, recent advancement in nanoscale imaging technologies, such as atomic force microscopy, may provide valuable insights into the molecular and mechanical landscapes of TME interactions and tumor progression [38]. Tumor-derived TGFβ and IL-6 have been shown to facilitate normal prostate fibroblast-to-myofibroblast transition [39,40]. Moreover, increased TGFβ also drives endothelial–mesenchymal transition and can lead to cell morphological changes that provide an alternative source of CAFs. TGFβ induces endothelial cells to express high levels of FSP-1 (fibroblast-specific protein-1), loosen cell–cell junctions, gain invasive characteristics, and promote tumor metastasis via CCL5 [41,42].

TGFβ or CXCL16/CXCR6-mediated chemotaxis have also been shown to induce tumor-directed trafficking and CAF-differentiation of bone marrow-derived MSCs [43]. Upon entry into the stroma, MSCs display a CAF-like phenotype and express α-SMA, Platelet-Derived Growth Factor Receptor Beta (PDGFRβ), and Fibroblast Activation Protein (FAP) [41]. MSC-derived CAFs have demonstrated perform vascular mimicry by attracting circulating monocytes to the tumor, and drive M2 macrophage polarization via TGFβ secretion [41]. This process increases blood supply and opens migration routes for invasive tumor cells. Thus, CAF-like MSCs have been shown to enhance tumor invasiveness and contribute to a heterogenous CAF population.

Importantly, many of the tumor-derived factors that drive the fibroblast-to-CAF transition, like TGFβ and IL-6, are also secreted by CAFs in later stages of PCa. Their purported roles in treatment resistance and potential utility in developing novel targeted therapies are discussed later in this review.

### 3.2. CAF Molecular Subtypes and Heterogeneity

Tumor resident fibroblasts generate a diverse pool of tissue-specific and tissue-resident CAF subsets with variable and overlapping protein and gene expression signatures. Therefore, CAF characterization has been largely based on cellular morphology, tissue localization, and exclusion criteria marking the absence of epithelial, endothelial, hematopoietic lineage markers, and genomic tumor signatures. FAP, FSP-1, vimentin, PDGFRs, and α-SMA are among the most dominant protein biomarkers overexpressed in CAFs compared to matched non-cancer-associated normal prostate fibroblasts [11]. However, none of these markers are exclusive to CAFs and are commonly expressed by other cell types within the TME [23,44]. Notably, the co-expression of multiple biomarkers indicates that diverse mechanisms contribute to the formation of activated fibroblasts within the TME.

Functional classification in solid tumors including breast, pancreatic, and colorectal cancer has defined three main distinct CAF subgroups: myofibroblastic CAFs (myCAFs), antigen-presenting CAFs (apCAFs), and inflammatory CAFs (iCAFs) [9,45]. These CAF signatures have also been identified in PCa tissue, indicating similarity in CAFs functions across different solid tumor types.

MyCAFs were initially described in PDAC, where they co-localize with tumor cells, express high levels of α-SMA, and are stimulated by TGFβ [46]. In PCa, myCAFs were shown to highly express activated fibroblast markers (*ACTA2*, *TAGLN*), ECM (*MYL9*, *TPM*) and collagen (*COL1A1*, *COL1A2*)-related genes, and demonstrated roles related to ECM remodeling, collagen metabolic processes, cell adhesion, and smooth muscle contraction [45]. ScRNA-seq analysis of seven primary CAF cultures found that myCAFs were enriched in three out of seven distinct CAF clusters that were associated with driver genes related to ECM organization and contractility (Cluster #1 *FN1*, *SERPINE1*, *GREM1*, *DSP*), adhesion (Cluster #4 *ITGA8*, *LGALS1*) and cytoskeletal organization (Cluster #6 *MACF1*, *FNDC3B*, *PTPRK*, *DENND2A*) [10]. Androgen deprivation has also been shown to correlate with the emergence of SPP1^+^ myCAFs, characterized by elevated *ACTA2* expression and ECM remodeling activity, suggesting that stromal contractile reprogramming contributes to tumor progression under ADT-related therapeutic stress [47].

The role of apCAFs in solid tumors remains somewhat controversial. Some studies reported that MHCII^+^ apCAFs promoted immune tolerance in breast and pancreatic cancer TMEs [48,49], while others suggested that apCAFs might bolster tumor immunity and could potentially be leveraged to enhance vaccine strategies in lung cancer [50]. ScRNA-seq analysis of 16 prostatectomy specimens from three independent cohorts captured an apCAF signature that was associated with increased expression of antigen presentation-related genes (*CD74*, *HLA-DRB1*, *HLA-DRA*) [45]. In an independent analysis of patient-derived CAFs, apCAF signatures (*SLPI*, *CD74*) associated with immune regulatory and antigen presentation pathways with considerable inter-patient heterogeneity [10]. Among the 7 distinct CAF clusters captured in this cohort, Cluster #7 associated with enriched apCAF signatures with *CD83* among the most differentially expressed (DE) genes. Moreover, scRNA-seq analysis of 12 prostatectomy specimens identified 20 apCAF- (referred to as Antigen Presentation and Process-related CAFs; APPCAFs) associated DE genes that showed prognostic value for biochemical recurrence (BCR); 4 of these genes demonstrated utility in constructing an apCAF-based prognostic risk signature (*THBS2*, *COL5A1*, *MARCKS*, *DPT*) [9]. Although there is growing evidence of apCAFs playing a relevant role in TIME (tumor immune microenvironment) regulation in PCa with potential translational value, their function and utility is yet to be specifically investigated.

CAFs secrete a large palette of paracrine immune regulatory factors, including chemokines and cytokines, which exert a potent role in shaping the TIME. CAF-derived IL-6, IL-8, CXCL1, CXCL12, hepatocyte growth factor (HGF), and TGFβ, recruit M2-like macrophages, myeloid-derived suppressor cells (MDSCs), and regulatory T cells (T_reg_) and collectively enrich an immunosuppressive milieu [11,51,52,53]. This subset of secretory CAFs is often referred to as “inflammatory” or “immunoregulatory” CAFs (iCAFs). In PCa, iCAFs have been shown to express inflammatory (*CXCL12*, *IGF1*) and complement-regulatory genes (*C3*, *C7*, and *CFD*). Similarly to myCAFs, iCAFs express ECM remodeling matrix metalloproteases (MMPs), MMP2, MMP14 [45]. Distinct iCAF subpopulations have been shown to recruit discrete immune cell populations, such as M1-like macrophages or plasma cells reflecting on multi-directional TIME interactions. Additionally, CAF signatures have been shown to associate with expression of negative checkpoint molecules and the sensitivity of tumor cells to CTLA-4 and PD-1 targeted immunotherapies, leading to considerable inter-patient variability in treatment efficacy [54]. These functional iCAF programs have also been recapitulated in physiologically relevant 3D PCa models. In a recent study, 3D organoid and *in vivo* systems identified two primary CAF programs: ECM-CAF and Lym-CAF. Lym-CAFs enhanced anti-tumor CD8^+^ T cell immunity, promoted immunotherapy responses, and partially overlapped with classic iCAFs. Mechanistically, YAP1 inhibition shifted CAFs toward the Lym-CAF state by inhibiting the IKK–NF–κB axis [55].

Initial studies of the PCa CAF pool revealed remarkable heterogeneity reflected in both origins and functions. As state-of-the-art molecular studies such as lineage tracing, scRNA-seq, and spatial transcriptomics have become more accessible, analyses of distinct molecular subpopulations have improved investigation of PCa-associated CAFs. This technological advancement may aid discovery of translationally relevant molecular subsets, beyond the major functional subcategories [56,57,58].

ScRNA-seq allows high-resolution insights into the complex molecular landscapes of heterogeneous niches, such as the PCa TME, in the context of inter-patient heterogeneity. Analysis of patient-derived CAF pools in prostatectomy specimens by Vickman et al. captured six distinct CAF clusters (Cluster 0–Cluster 5; C0–C5) [56]. The top DE genes for Clusters C0 to C5 were *GLRX*, *PKM*, *CD63*, *BIRC5*, *PHKG1* and *MALAT1*, respectively, with the majority of CAFs rendered to two main clusters, C0 and C1. While overexpression of *ACTA2*, *FAP*, and *DPP4* (CD26) showed considerable heterogeneity across the distinct CAF subsets, *VIM* and *THY1* (CD90) were expressed more uniformly. CD90 overexpression has been captured across different CAF subsets. CD90 is known to promote tumorigenesis through TGFβ, hedgehog signaling and other immune regulatory pathways [59]. Additionally, CD90^+^/CXCL12^+^ CAFs have been found to protect BPH-1 cells from hydrogen peroxide-induced apoptosis, suggesting a potential role in promoting prostate epithelial cell survival [59]. CD90 is known to play a versatile role in regulating prostate tissue homeostasis as a costimulatory molecule on both epithelial and immune cells. Moreover, increased CD90 immunostaining on fibromuscular cells within prostate tumor foci versus adjacent benign tissue was proposed as a potential biomarker for tumor-associated mesenchymal changes that sharply delineate tumor from surrounding normal tissue [60].

Investigation into the functions of distinct CAF clusters by Vickman et al. [56] revealed specific chemokine/cytokine-related genes among the DE genes (*CCL11*, *CXCL12*, *CXCL1*, *IL33*). *CCL2* and *CXCL12* were the most predominantly expressed in clusters C0 and C1, respectively. These specific CAF-derived chemokines play a significant role in driving inflammatory pathways, immune infiltration and myeloid cell recruitment. Therefore, clusters C0 and C1 could have an important role in reshaping localized TIME neighborhoods to foster immune privileged niches.

Multi-analyte molecular profiling of 26 hormone-sensitive and 8 castration-resistant PCa samples [61] identified eight CAF subsets with two dominant subtypes, *α-SMA*^+^*CAV1*^+^ CAFs (CAFs-C0) and *FN1*^+^*FAP*^+^ CAFs (CAFs-C1). Protein biomarker analysis validated these two main subsets showing α-SMA/CAV1 co-expression and FN1/FAP co-expression on distinct CAF cells, with a negligible overlap of α-SMA and FN1 binding. Furthermore, CAFs-C0 exhibited high expression of several microvascular genes (*MYH1*, *MCAM*, *RGS5*), and pathway enrichment analysis showed associations to microvascular development. In contrast, the CAF-C1 subtype showed elevated levels of decorin and various collagen molecules, and enrichment of ECM organization pathways. Notably, survival analysis in TCGA-PRAD and GSE116918 cohorts showed that CAF-C1 was linked to poor prognosis, castration resistance, and reduced response to immune checkpoint inhibitor therapy.

While there are several common hallmark biomarker patterns captured by these scRNA-seq studies across independent patient datasets, the considerable heterogeneity detected in the molecular CAF landscapes emphasizes the need for future investigation on expanded clinical cohorts.

A recent pan cancer study from 2023 utilized a comprehensive single-cell transcriptomic atlas across 12 solid tumor types representing 210 patients, including 6 neuroendocrine prostate cancer (NEPC) [62] and 20 prostate adenocarcinoma cases (PRAD) [57]. This report identified four distinct CAF clusters [63]: progenitor CAF (proCAF), inflammatory CAF (iCAF), myofibroblastic CAF (myCAF), and matrix-producing CAF (matCAF). The proCAF subtype was characterized by the expression of *IGF1*, *OGN*, and *C7*. MyCAFs exhibited high levels of *RGS5*, *MYH11*, and *ACTA2*, while matCAFs were defined by the expression of *COL10A1*, *CTHRC1*, and *POSTN.* The iCAF population associated with elevated levels of *CCL2*, *CXCL12*, and *CXCL14*. ProCAFs appeared to share functions with different CAF subtypes, suggesting this subset may serve as a key source for various CAF subcategories. The matCAF signature is associated with the initiation and progression of cancer and has been linked to poor prognosis in several cancer types.

Notably, the myCAF signature was dominant in the PCa samples in this study with limited presence of iCAFs, while the matCAF and proCAF subtypes were largely absent. The authors noted that the sample size was limiting and further research was needed to investigate the potential role of proCAFs and matCAFs in PCa pathogenesis. However, a study in a 3D PCa organoid model identified a matCAF-like subset, ‘ECM-CAF’ that promoted ECM and collagen deposition and supported tumor progression in a YAP1 dependent manner [55].

There is growing evidence suggesting that CAFs may play an influential role in shaping heterogenous clinical trajectories in PCa. Integrated scRNA-seq and bulk RNA-seq data from PCa patients in TCGA was used to develop a CAF-related gene prognostic index (CRGPI) based on a set of eight CAF-related genes *(NDRG2*, *TSPAN1*, *PTN*, *APOE*, *OR51E2*, *P4HB*, *STEAP1*, *ABCC4*) that correlated with prognosis [64]. The CRGPI was further validated in two additional study cohorts (MSKCC2010;GSE46602). Subtype 1 in the TCGA cohort associated with a higher median CRGPI score and poor prognosis, while subtype 2 was linked to lessened risk of BCR [64,65,66,67].

CAFs exert remarkably versatile roles in shaping TME architecture and interactions in co-localized cellular networks, contributing to intra-foci heterogeneity. Therefore, understanding the role of CAF in the context of spatial organization within prostate tumors is essential. Single cell spatial transcriptomic profiling using the CosMx and MERISCOPE platforms investigated CAF landscapes in eight solid tumor types including PCa [68]. This study identified four major CAF subtypes with distinct DE gene expression profiles, s1- to s4-CAF. The s1-CAF profile mostly resembled previously defined myCAF signatures with hallmark genes of ECM remodeling, collagens, MMPs and smooth muscle contraction. Meanwhile, s2-CAFs associated with inflammation and immune suppression, similarly, to previously established FAP^+^α-SMA^+^ CAF profiles. The s3-CAF signature shared iCAF and apCAFs biomarkers, including genes that are associated with stress response, antigen presentation and PDGFRs. The s4-CAF subset was most highly expressing apCAF genes related to antigen presentation, cytokines and STAT3, but also shared iCAF-like chemokine profiles. Ligand-receptor analysis suggested that s1-CAFs predominantly interacted with tumor cells through ECM molecules, integrins, and the TGFβ1 and FGF2 axes, while s2-CAF mostly engaged with endothelial cells and stroma-infiltrating T cells. S3-CAF profiles co-localized with myeloid components with connections to macrophages and neutrophils, and s4-CAFs co-localized to lymphoid aggregates and tertiary lymphoid structures including T cells, B cells and plasma cells. The study concluded that these spatial subtypes and neighborhood organization features were largely conserved across various solid tumor types.

Collectively, the findings in recent state-of-the-art molecular studies have provided new insights into CAF granularity. They have also underscored the persistent challenge in classifying heterogeneous CAF subpopulations and defining hallmark biomarker features. However, further research is needed to clarify the specific role and translational value of various molecular CAF subtypes in tumor progression and identify factors that drive their recruitment. Future investigation of the TME interactome by spatial molecular profiling in longitudinal investigations may expedite such discovery.

Altogether, better understandings of these factors will lay the foundation for developing improved CAF-targeted therapeutic strategies that exploit tumor-promoting regulatory pathways and disrupt the tumorigenic TME [61]. High-density, heterogeneous populations of CAFs have been previously associated with poor clinical outcomes and transcriptomic analysis showed that CAFs significantly associate with BCR-free survival [64]. Therefore, investigation of the prognostic value of specific molecular CAF subtypes may yield valuable insight into the specific CAF functions that support the development of TME-targeted therapies to control high-risk disease.

### 3.3. The Role of CAFs in the Prostate TME

**Figure 1:**
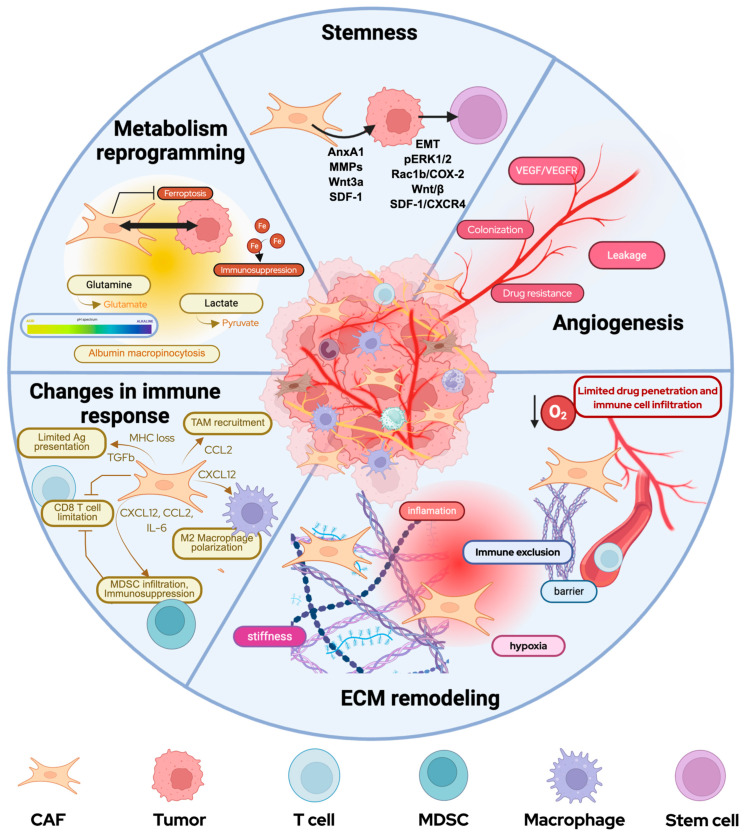
**CAF functions in prostate cancer.** CAFs are a versatile cellular subset that play important roles in multiple relevant functions of the TME that promote tumor growth and resistance through regulation of angiogenesis, ECM remodeling, immune functions, metabolic reprogramming and enhancing stemness. Arrows: Activation or secretion, Inhibitory arrow: Inhibition, ↓: Downregulation. Created in Biorender.com.

CAFs are a multifaceted TME component that mediate various functions to either promote or suppress tumor formation (Figure 1). The pro-tumorigenic functions encompass the promotion of angiogenesis, epithelial cell proliferation, and an immunosuppressive TME [22,69,70]. Tumor cells and CAFs interact via paracrine reciprocal feedback loops involving cytokines, growth factors, and extracellular vesicles. These promote tumor growth, cell migration, ECM remodeling, and alterations in the TME [11,71,72]. The CAF-tumor co-evolution depends on factors such as primary tumor type, intrinsic tumor features, proliferation rate, and stromal composition [72]. Within this section, we discuss the latest advances in this field that reveal the diverse roles of CAFs in prostate tumorigenesis and tumor progression.

#### 3.3.1. ECM Remodeling

The ECM is the non-cellular structure in all tissues [73] that provides a mechanical scaffold. Additionally, the ECM serves as a reservoir for growth factors, cytokines, and chemokines that modulate cellular activities in specialized microenvironments. Prostate ECM is rich in structural proteins such as collagens, glycoproteins, laminins, fibronectins, and tenascins [74,75,76]. Prostate tumor cells promote aberrant ECM deposition and tissue remodeling while CAFs secrete excess amounts of tenascin, periostin, collagen, and fibronectin to collectively support tumorigenic ECM adaptations [11]. While fibroblasts are amajor source of ECM, they also produce enzymes that modify, degrade, and reorganize the structural molecules, thus playing an essential role in ECM formation, turnover, and remodeling. CAF-derived proteases, such as MMPs, drive hydrolysis, cross-linking, assembly, and fiber reorganization [77]. Consistent with these mechanisms, tumor–fibroblast co-culture studies in 3D model systems also showed significant ECM remodeling, including accumulation and MMP-driven proteolytic processing of basement membrane proteins as well as increased invasive tumor cell behavior, underscoring the importance of tissue-like architecture for studying stromal effects [78].

Integrated analysis of collagen fiber alignment and organization by second-harmonic generation (SHG) microscopy of 30 prostatectomy specimens revealed a significant differential in collagen alignment between tumor foci and normal adjacent areas. Collagen fiber cross-linking and orientation (measured as ratio of number of areas with strongly aligned fibers versus areas with randomly oriented fibers) was significantly higher in tumor versus normal tissue and positively correlated with Gleason score [79,80,81]. Stromal ECM regulators such as the proteoglycan decorin, likely produced by CAFs, are required for proper collagen organization and influence the collagen architecture [79]. The tissue remodeling activity of CAFs alters the ECM architecture and stiffness, which generates subsequent mechanical stresses that modulate tumor cell proliferation and oxygen distribution, thereby promoting adaptation to hypoxia and tumor progression [25,27,81,82,83,84,85,86,87]. These mechano-transduction signals generated by the rigid, desmoplastic ECM also further amplify CAF activation and ECM production and create a loop between stromal fibrosis, angiogenesis, and PCa progression.

Desmoplasia is a form of tumor-associated fibrosis characterized by the growth of dense, collagen-rich fibrous tissue around the tumor that develops in response to tumorigenesis. CAF-derived TGFβ signaling contributes to the formation of a dense ECM containing collagen I, fibronectin, proteoglycan syndecan-1, hyaluronic acid and tenascin-C [26]. Molecular analysis of heterocellular epithelial–mesenchymal–stromal tumoroids showed TGFβ-driven matrix remodeling and desmoplastic ECM changes which contributed to EMT and the development of an invasive phenotype. This suggests a functional link between TGFβ-driven fibrosis and progression in a 3D environment [88].

TGFβ has been shown to induce translocation of SMAD2/3 to the nucleus of PCa cells, amplifying their expression of mesenchymal markers, leading to EMT, and favoring PCa progression [88]. Furthermore, desmoplasia creates a physical barrier around the tumor, limiting drug delivery and driving treatment resistance [26]. This physical barrier around the tumor nest inhibits immune cell infiltration, which limits anti-tumor immune responses and promotes an overall “cold” TIME in PCa and other cancers. CAF-driven immune exclusion has been assessed as a potentially targetable mechanism to restore immune recruitment, anti-tumor T cell response and enhance immunotherapy efficacy [89,90].

#### 3.3.2. Angiogenesis

CAF-induced ECM remodeling and desmoplasia also cause compression of the tumor microvascular system and increase vascular density in the surrounding stroma. Furthermore, CAFs have been shown to upregulate hypoxia-induced angiogenesis regulator (HIAR) expression, which induces the VEGF/VEGFR signaling pathway in endothelial cells and promotes angiogenesis, contributing to tumor growth and drug resistance. CAFs can also be a direct source of VEGF-A, which induces lymphatic endothelial cell permeability and creates pre-conditioned, vessel-rich pre-metastatic microenvironments to promote colonization [91,92]. To investigate whether stromal compartments can drive pro-angiogenic signaling, a heterogeneous cancer–stromal sphere (CSS) co-culture model was created with PC3 cells and MSCs. CSS-conditioned medium increased VEGFA levels in co-cultures compared to tumor spheroid mono-cultures, and RNA sequencing confirmed the stromal origin of VEGFA [93].

CAFs in PCa frequently upregulate PDGFRs, which mediate stromal–endothelial interactions and modulate endothelial cell behavior and vascular permeability. In a study of 300+ TURP (Transurethral Resection of the Prostate) tissue specimens, including metastatic and non-metastatic cases, high stromal and perivascular PDGFRβ protein expression was linked to higher Gleason score [94]. In a multi-center study of 535 prostatectomy specimens, high stroma-derived expression of PDGFRβ was associated with poor clinical outcomes including BCR and early clinical relapse. Therefore, PDGFRβ was proposed as a potential prognostic marker for stratification of high-risk disease [95].

Using 3D *in vitro* models, PCa-conditioned media was shown to induce reprogramming of bone-derived fibroblasts into a MAF/CAF state, which was associated with changes in the expression of N-cadherin, non-functional E-cadherin, α-SMA, tenascin C, vimentin, TGFβ R1 and R2. Transcriptomic analysis revealed enhancement of pathways regulating cancer invasion, proliferation, and angiogenesis [96].

In summary, there is growing evidence suggesting that the complex role of CAFs in angiogenesis promotes PCa growth and may provide a potentially exploitable pathway to control disease progression.

#### 3.3.3. CAFs Contribution to Enhancing Tumor Stemness

Stemness in tumor cells associates with enhancement of properties such as self-renewal, immortalization, de-differentiation, and efflux of drugs. Stem-like molecular signatures have been associated with aggressive forms of PCa and poor clinical trajectories including the development of metastasis, CRPC, and neuroendocrine differentiation [97,98].

The role of CAFs in promoting stem transformation has been established in several solid tumors, including colorectal, breast, or lung cancer [99,100,101]. In a conditional PTEN deletion mouse model of PCa, it was discovered that CAF-derived secreted factor AnxA1 promoted the stemness and growth of cancer stem-like cells [102]. Treatment of tumor cells with AnxA1 was found to upregulate epithelial–mesenchymal transition (EMT) and pluripotency factors in vitro. Additionally, AnxA1 treatment led to enhanced formation of glandular structures, increased expression of p63 *in vivo*, and was associated with the activation of pErk1/2 both *in vivo* and *in vitro*. These findings suggest that CAF-derived AnxA1 may play a role in the generation and maintenance of PCa cancer stem cells (CSCs) by promoting epithelial cell de-differentiation into CSCs, and enhancing proliferation and differentiation of CSCs [102]. In spheroid co-cultures, CAF-derived conditioned media enhanced tumor cell expression of hallmark stem cell biomarkers (*SOX2*, *OCT4*, *Nanog*) [51,102]. Co-implantation of CAF versus normal prostate fibroblasts with CSCs into a PTEN^−/−^ host showed that CAFs promoted the growth of larger spheroids with enhanced glandular structures, further supporting that CAFs enhance both the stemness and growth potential of CSCs [103]. Similarly, a CAF-conditioned media from 3D prostaspheres and primary human CAFs induced EMT and stem cell-like properties such as increasing CD44^high^/CD24^low^ and CD133^+^ cells in the PCa subset. This was mediated by an inflammatory–oxidative axis (NF-κB, COX-2, HIF-1α, ROS). Inhibiting these factors blocked CD133^+^ expansion, reduced EMT markers, and suppressed prostasphere formation. The findings further demonstrate that CAFs promote a CSC-like phenotype in PCa cells via EMT.

Furthermore, CAF-secreted MMPs promote EMT and stem-cell traits in PCa cells by inducing tumor-cell release of reactive oxygen species via a Rac1b/COX-2 axis [104,105]. During CRPC progression, CAFs promote CSC properties by secreting Wnt3a and SDF-1 (also known as CXCL12), which activate the Wnt/β-catenin and SDF-1/CXCR4 signaling pathways [105]. The CXCL12-CXCR4 axis has also been implicated in promoting invasiveness, MMP secretion and migratory capacity of prostate tumor cells [106].

#### 3.3.4. Reprogramming of Cancer Metabolism

Studies of PCa pathogenesis have demonstrated that CAFs promote tumor progression by reprogramming cancer metabolism. CAFs and tumor cells form a symbiotic relationship within the TME ecosystem and undergo reciprocal metabolic reprogramming [107]. Specifically, under contact with tumor cells, CAF metabolism shifts from oxidative phosphorylation to aerobic glycolysis [108]. Tumor cells hijack regulation of the CAF mitochondrial metabolism, which leads to increased lactate production in CAFs [107]. The resulting switch of cancer cells to oxidative phosphorylation and of CAFs to aerobic glycolysis is referred to as the “reverse Warburg effect” [109]. Lactate secreted from CAFs serves as an alternative fuel source for oxidative tumor cells, which convert lactate to pyruvate via lactate dehydrogenase, driving the tricarboxylic acid cycle and oxidative phosphorylation, thereby supporting cancer cell survival and metabolic flexibility under nutrient-limited or moderately hypoxic conditions [107,108,110,111]. Additionally, lactate release decreases the pH of the microenvironment and influences the immune infiltrate of the TME. Specifically, the acidic environment impairs cytotoxic T cell responses and promotes M2-like macrophage polarization, which further contributes to tumor evasion [112,113,114,115].

Glutamine is an essential amino acid and serves as a primary nitrogen and energy source for tumor cells. During glutaminolysis, glutamine is converted to glutamate [116] and elevated glutamate serum level has been previously associated with higher Gleason scores in PCa [117]. CAFs make significant contributions to the TME glutamine pool, providing an alternative metabolic source and facilitating tumor growth. Excessive tumor-induced RAS activation in PCa-associated CAFs have also been shown to induce macropinocytosis-driven albumin uptake and glutamine production, which in turn provide an alternative glutamine-related metabolic feedback loop for tumor cells [118].

Iron accumulating CAFs, also known as FerroCAFs, were identified in human PCa tissue and were shown to enrich in tumor foci with higher Gleason scores versus normal adjacent or BPH tissue. FerroCAF infiltrates were also found in mCRPC tissue biopsies suggesting links to PCa progression. ScRNA-seq analysis of a previously published dataset of 13 PCa tumor samples [57] established a FerroCAF signature based on *HMOX1*, *KDM6B*, *CCL2*, *CSF1*, *IL6*, *CXCL1* and *CXCL2* overexpression, and found FerroCAF enrichment in high Gleason score tumors [119]. Hmox1-mediated accumulation of intracellular iron activates the epigenetic regulator Kdm6b, which induces immunosuppressive cytokine and chemokine expression, leading to myeloid cells recruitment and T cell suppression [119]. Gene Set Variation Analysis (GSVA) of this scRNA-seq data set established a FerroScore based on the *HMOX1*, *KDM6B*, *PVR*, *CCL2*, *CSF1*, *IL6*, *CXCL1* gene set and revealed associations with stromal signatures and accumulation in the CAF pool. Moreover, GSVA found an association between the FerroScore and shorter overall survival in the TCGA PCa data set and positive correlation with BCR in the GSE46602 cohort, suggesting a linkage of FerroCAF enrichment with poor clinical outcomes [119].

In a 3D models system, CAFs were shown to induce cholesterol and steroid metabolic pathways in LNCaP and DuCaP spheroids. This specifically increased *HMGCS2* and *AKR1C3* expression and associated with the development of anti-androgen resistance [120].

#### 3.3.5. Changes in the Immune Response

PCa has been associated with a differential immune infiltrate compared to healthy prostate tissue; this results in changes to how both innate and adaptive immune cells interact with tumor cells and CAFs to shape tumor behavior and therapeutic response [91,121]. Immunotherapies (e.g., PD-1/CTLA-4 inhibitors, adoptive cell therapies) have led to breakthroughs in several solid tumors, though their effect has been limited in PCa. The prostate TME is considered a cold immune microenvironment due to multiple factors including a low tumor mutation burden, epigenetic loss of MHC I, MHC II and related antigen-presenting machinery, immune suppressive cell subsets, limited T cell trafficking and immune surveillance [122,123,124,125]. Immune evasion is a hallmark of malignant progression in PCa [126,127] and CAFs have also been implicated in this by recruiting a heterogenous pool of suppressive immune subsets that enforce chemokine barriers. PCa-associated CAFs recruit tumor-associated macrophages (TAMs) via CCL2 secretion [59]. CAF-derived CXCL12 production also promotes M2-like macrophage polarization, which has been associated with poor clinical outcomes in PCa [80,128]. CAF-derived CXCL12 also contributes to the recruitment and activation of CXCR4^+^ mast cells, promoting inflammatory and pro-tumorigenic signaling within the TME [56]. Additionally, immune evasion is exacerbated by CAF-driven immune exclusion enforced by ECM deposits that raise a physical barrier around the tumor nest and limit tumor-specific T cell trafficking [129,130].

In the previously described CSS model, conditioned medium from PCa-MSC co-cultures induced the stroma-associated immunoregulatory factors VEGFA, IL-10, and IL1a. IL-10 inhibits anti-tumor T cell response and promotes M2 polarization while VEGFA may contribute to immune exclusion by forming abnormal vascular networks. Thus, the CSS model provided further evidence that stroma promoted an immunosuppressive TME [93].

In a 3D prostate ductal model in the LumeNEXT platform comparing non-tumorigenic and metastatic TMEs prostate epithelial lumens were co-cultured with primary CAFs and immune cells. Metabolic and cytokine profiles of immune cells reflected a differential response in the metastatic setting including increased GM-CSF, IL1 secretion and reduced STAT1, STAT3 and TLR2, TLR4 signaling; this provided further evidence that spatial interactions between tumor cells and CAFs contributed to an immune suppressive milieu in the PCa [131].

Both directly and indirectly, CAFs play a pivotal role in mediating diverse immune functions by integrating multiple signaling networks within the TME that affect interactions across multiple axes, between tumor, stroma, and epithelial components and dynamically change throughout tumor development. Immune evasion and immunosuppressive features are considered hallmarks of the PCa TME that foster tumor progression, and immune-related biomarkers have been correlated with PCa clinical outcomes, indicating potential prognostic value. Therefore, leveraging specific biomarkers and targetable molecular pathways in the CAF–immune axis may allow exploitation of CAF-mediated tumor regulatory functions, enhance anti-tumor immune activity, and facilitate response to immunotherapies [132,133,134].

## 4. CAF Contribution to Therapy Resistance

### 4.1. Tumor Targeted Therapies

#### 4.1.1. CAF Contribution in AR-Directed Therapy Resistance

In advanced PCa, ADT and ARPIs are first-line standard of care therapies and are often given in combination [135,136]. The most commonly prescribed FDA-approved AR-directed therapies are abiraterone acetate (inhibiting androgen biosynthesis), enzalutamide, bicalutamide, apalutamide, and darolutamide (AR antagonists). Although these therapies have been associated with significant clinical benefit, treatment resistance is inevitable [137]. While AR genomic alterations such as AR amplification or AR splice variants [138,139] are putative mechanisms of resistance, the exact factors and mechanisms driving tumor plasticity and genomic tumor evolution in response to AR targeted therapies are not clearly understood [140]. However, prostate fibroblasts have been shown to induce AR-V7 expression in PCa cells [141] via IL-8 secretion. Additionally, AR-V7 has been found to be expressed in primary CAFs isolated from prostatectomy specimens [142], and ARSI-induced IL-6 facilitated CD105/BMP (bone morphogenic protein) and RMB38-mediated RNA splicing in both cell types to generate AR-V7. Consequently, cell death in PCa cells in response to ARSI treatment was attenuated after 3D co-culture with AR-V7 overexpressing fibroblasts, providing strong rationale for further investigation on how the CAF-tumor axes may be exploited in development of ARSI re-sensitization strategies.

Accordingly, AR signaling pathway is active in both CAFs and normal fibroblasts, including myofibroblasts, rendering them sensitive to changes in tumor androgen levels [143,144]. ChIP-seq studies found that the AR cistrome in human myofibroblasts was distinct from PCa cell line AR cistromes, with a greater dependence on AP-1 as a pioneer factor in fibroblasts, in contrast with FOXA1 in PCa cells [145,146]. Selective deletion of fibroblast AR led to abnormal prostate development and disruption of the stromal ECM in mice, suggesting an important role for stromal AR in prostate organogenesis [147]. Specifically, knockout of fibroblast AR reduced the expression of key growth factors, including IGF-1, FGF-7, and FGF-10 [147]. AR pathway inhibition in CAFs has induced similar changes in expression of several CAF-derived paracrine factors, leading to the development of a pro-tumorigenic microenvironment that promotes ARPI resistance in tumor cells. For example, increased CAF-derived secretion of CCL2 and CXCL8 in response to AR signaling inhibition in CAFs was associated with PCa invasiveness [148]. In a genetically engineered mouse model (GEMM) of PCa, anti-androgen treatment affected both the tumor and stromal compartment. After ADT treatment, activated TGFβ signaling in CAFs induced a phenotypic shift in iCAFs to SPP1^+^ myCAFs through a SOX4-SWI/SNF-dependent mechanism, and these activated myCAFs subsequently contributed to ADT resistance through paracrine SPP1–ERK signaling [47].

Elevated IL-6 production within the TME has also been shown to promote ligand-independent AR transcriptional activity via STAT3 and MAPK signaling, whereas PI3K/AKT signaling can suppress IL-6 driven AR transactivation [149]. Moreover, after ADT treatment, LIM domain only 2 (LMO2), which is repressed by AR in normal, healthy prostate fibroblasts, was upregulated in immortalized PCa fibroblasts (WPMY-1). ADT-induced overexpression of LMO2 in CAFs caused paracrine release of FGF-9 and IL-11, driving tumor cell growth and castration resistance. Analysis of the TCGA PRAD dataset revealed a link between elevated IL-11 and IL11R expression in PCa tissue and shortened disease-free survival. Furthermore, serum IL-11 levels were increased in metastatic disease versus primary tumors or benign prostates. Serum IL-11 levels in this cohort were predictive for PCa metastasis, with predictive value comparable of serum PSA [150]. Furthermore, LMO2 expression is often higher in aggressive PCa tumor cells [151] and it has been shown to downregulate E-cadherin expression and boost motility of PCa cells in vitro [152].

Lastly, AR pathway inhibition can induce epigenetic changes in CAFs, fostering a metabolic niche for tumor cells to adapt to AR blockade. For example, enzalutamide and bicalutamide induced promoter hypermethylation of the RAS inhibitor RASAL3 gene, driving oncogenic Ras activity and macropinocytosis-mediated albumin uptake [118]. Lysosomal degradation of albumin in Ras-activated CAFs is linked to increased glutamine secretion. Subsequently, glutamine uptake by tumor cells could contribute to tricarboxylic acid cycle anaplerosis and mTOR activation, which has been linked to neuroendocrine differentiation [118].

Together, these studies highlight mechanisms through which AR-targeted therapies causing CAF AR inhibition may contribute to resistance in tumor cells. While the role of stromal AR activity in clinical prognosis and disease trajectories in advanced PCa has been controversial, loss of stromal AR signaling has been associated with reduced overall survival and higher Gleason score [144,153,154,155,156]. The complex functional role of CAFs in the development of ARPI resistance predicts potential utility of combinatory targeting of both epithelial and CAF AR pathway activity in delaying the development of CRPC and differentiation of aggressive forms of PCa.

**Figure 2:**
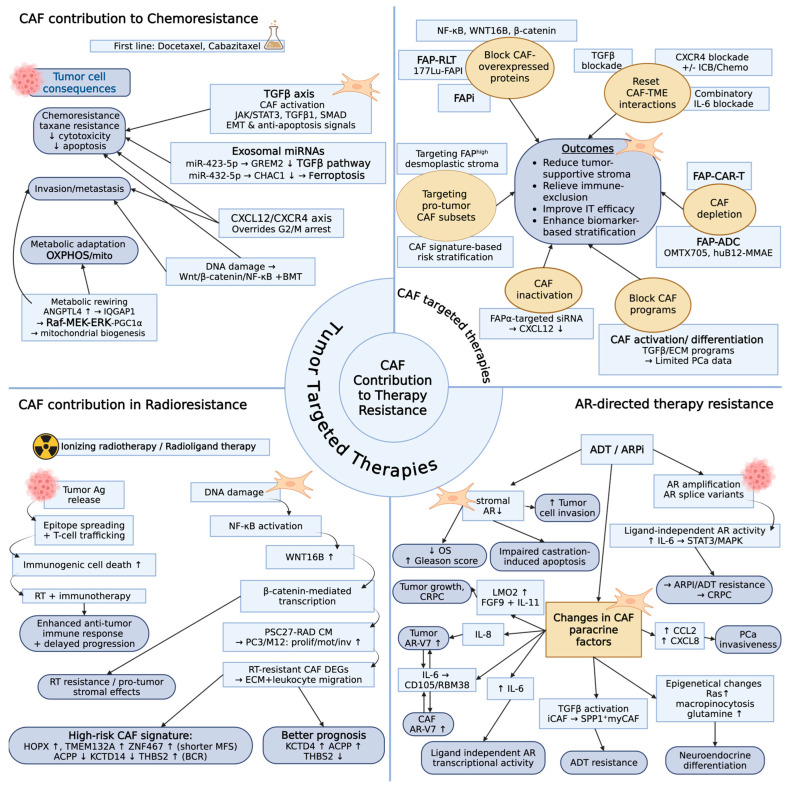
**CAF contribution to therapy resistance and CAF-targeting strategies.** Schematic overview of CAF-derived mechanisms that promote PCa resistance to chemotherapy, radiotherapy, and AR-directed therapy. **Cells, tumor biology, and microenvironment:** CAF, Cancer-Associated Fibroblast; TME, Tumor Microenvironment; iCAF, Inflammatory Cancer-Associated Fibroblast; myCAF, Myofibroblastic Cancer-Associated Fibroblast; CRPC, Castration-Resistant Prostate Cancer; PCa, Prostate Cancer. **Therapies and treatment modalities:** ADT, Androgen Deprivation Therapy; ARPi, Androgen Receptor Pathway Inhibitor; ICB, Immune Checkpoint Blockade; RT, Radiotherapy; CAR-T, Chimeric Antigen Receptor T-cell Therapy; ADC, Antibody–Drug Conjugate; siRNA, Small Interfering RNA. **Receptors, ligands, and signaling pathways:** TGFβ, Transforming Growth Factor Beta; AR, Androgen Receptor; NF-κB, Nuclear Factor Kappa B; WNT, Wnt Signaling Proteins; WNT16B, Wnt Family Member 16B; β-catenin, Beta-Catenin; CXCL12, C-X-C Motif Chemokine Ligand 12; CXCR4, C-X-C Motif Chemokine Receptor 4; IL-6, Interleukin-6; STAT3, Signal Transducer and Activator of Transcription 3; MAPK, Mitogen-Activated Protein Kinase; JAK/STAT, Janus Kinase/STAT Signaling Pathway; SMAD, SMAD Family Transcription Factors; EMT, Epithelial–Mesenchymal Transition; ECM, Extracellular Matrix. **Genes and proteins:** FAP, Fibroblast Activation Protein; FAPI, Fibroblast Activation Protein Inhibitor; GREM2, Gremlin 2; CHAC1, ChaC Glutathione-Specific Gamma-Glutamylcyclotransferase 1; ANGPTL4, Angiopoietin-Like 4; IQGAP1, IQ Motif Containing GTPase-Activating Protein 1; PGC1α, Peroxisome Proliferator-Activated Receptor Gamma Coactivator 1 Alpha; LMO2, LIM Domain Only Protein 2; FGF9, Fibroblast Growth Factor 9; SPP1, Secreted Phosphoprotein 1; RAS, RAS Proto-Oncogene Family; ZNF467, Zinc Finger Protein 467; TMEM132A, Transmembrane Protein 132A; HOPX, Homeodomain-Only Protein X; THBS2, Thrombospondin 2; KCTD4, Potassium Channel Tetramerization Domain Containing 4; ACPP, Acid Phosphatase, Prostate. **Metabolism, cell cycle, and cell death:** OXPHOS, Oxidative Phosphorylation; DNA, Deoxyribonucleic Acid; OS, Oxidative Stress or Overall Survival; G2/M, G2/M Phase of the Cell Cycle; Ag, Antigen. **Radiotherapy and radioligand therapy**: RLT, Radioligand Therapy; Lu, Lutetium; ^177^Lu-FAPI, Lutetium-177–Labeled Fibroblast Activation Protein Inhibitor; DEGs, Differentially Expressed Genes; PSMA, Prostate-Specific Membrane Antigen. **Clinical and experimental terms:** MFS, Metastasis-Free Survival; BCR, Biochemical Recurrence; CM, Conditioned Medium. **Drugs and constructs:** OMTX705, FAP-Targeting Antibody–Drug Conjugate; huB12-MMAE, Humanized B12 Antibody–Monomethyl Auristatin E; FAP-ADC, Fibroblast Activation Protein-Targeting Antibody–Drug Conjugate; FAP-CAR-T, Fibroblast Activation Protein-Targeting CAR-T Cell Therapy. Arrows: Activation or secretion, Inhibitory arrow: Inhibition, ↓: Downregulation, ↑: Upregulation, ↑↓: Interaction, →: Process. Created in Biorender.com.

Contribution of CAFs to therapy resistance and CAF-targeted therapies are summarized in Figure 2.

#### 4.1.2. CAF Contribution to Chemoresistance

Docetaxel and cabazitaxel chemotherapy are first-line therapies for advanced CRPC or metastatic disease [157]. Other chemotherapies used to treat mCRPC include cisplatin, carboplatin, and etoposide [158]. These systemic therapies may be prescribed as single agents or in combination to improve overall survival and/or quality of life. Like ARPI treatments, however, therapeutic resistance is inevitable though mechanisms of chemoresistance are less clear. CAFs have been shown to inhibit the effectiveness of chemotherapy in both preclinical and translational studies primarily via secreted factors.

Several paracrine signaling pathways have been implicated in CAF-driven chemoresistance. A fungal polysaccharide, MPSSS, has been previously shown to attenuate the immunosuppressive effects of CAFs [159] by suppressing CAF-derived TGFβ1 secretion [160] and increasing CAF chemosensitivity to docetaxel through counteracting CAF-promoted EMT and anti-apoptotic signals. Bioinformatic analysis on the GSE33455 and GSE158494 cohorts found TGFβ signaling to be the most enriched pathway in docetaxel-resistant PCa cells. Elevated CAF-derived TGFβ1 levels were associated with docetaxel resistance, which was abrogated by TGFβ1 receptor inhibition. Moreover, gene ontology (GO) enrichment analysis indicated activation of the SMAD complex and phospho-SMAD2, which could be reduced by MPSSS treatment. Mechanistic studies showed that MPSSS reduced CAF activation and JAK2/STAT3 pathway phosphorylation, thereby decreasing TGFβ1 production and chemoresistance via the TLR4 receptor.

CXCL12, a chemokine abundantly produced by CAFs, has been shown to play a significant role in tumor biology by activating the CXCR4 chemokine receptor on tumor cells; this signaling pathway had been implicated in CAF-driven chemoresistance. CXCL12 treatment was shown to rescue PCa cells from docetaxel-induced killing by overriding the microtubule stabilization-driven G2/M cell cycle arrest in addition to activation of LIM domain kinase 1 [161]. In the context of bone metastasis, CXCL12 was shown to facilitate the metastatic spread to bone by inducing adhesion, and migration of PCa cells through endothelial layers and invasion through basement membranes [162].

In a preclinical murine model of PCa bone metastasis, combination treatment of docetaxel and balixafortide, a CXCR4 inhibitor, reduced tumor burden and increased serum IFNγ levels in tumor-bearing mice, suggesting that inhibition of the CXCL12/CXCR4 axis may enhance docetaxel efficacy in bone [163].

Beyond chemokines and cytokines, other paracrine CAF-derived factors that may influence PCa chemoresistance have been investigated. CAF-derived exosomes carrying miR-423-5p were shown to decrease chemosensitivity to taxanes in LNCaP, 22Rv1, and C4-2 cells and further increased drug resistance in docetaxel-, taxane-, and bicalutamide-resistant variants by inhibiting GREM2 via the TGFβ pathway. Blockade of TGFβ reversed the CAF-exosome-derived drug resistance [164].

Alternatively, primary CAF-derived exosomal miR-432-5p was also found to reduce docetaxel sensitivity in PCa cells by reducing ferroptosis, a non-apoptotic, iron-dependent form of programmed cell death triggered by excessive accumulation of lipid peroxides [165]. This study showed that CAF-derived miR-432-5p diminished glutathione consumption by targeting CHAC1, an early marker of ferroptosis. ShRNA-mediated knock-down of CAF miR-432-5p enhanced docetaxel sensitivity in tumor-bearing mice. While the CAF–miR-432-5p–CHAC1–ferroptosis axis provides an alternative mechanism driving docetaxel resistance and a potential CAF-targeted approach to restore PCa chemosensitivity, miR-432-5p is widely expressed in normal tissues; therefore, further investigation is needed to develop translational utility of this approach.

Analysis of prostatectomy tissue after neoadjuvant mitoxantrone and docetaxel therapy found increased WNT16B expression and DNA damage in stromal fibroblasts. Similarly, mitoxantrone treatment of human prostate fibroblasts induced expression ofDNA damage response biomarkers, including WNT16B. Elevated stromal WNT16B expression was also found in prostatectomy tissue after therapy, which was linked to shortened biochemical relapse-free survival. Conditioned media from WNT16B-expressing fibroblasts promoted growth and invasiveness of metastatic PCa cell lines along with activation of Wnt signaling and enhancement of EMT characteristics, such as N-cadherin overexpression. *In vivo* co-implantation of WNT16B-expressing fibroblasts and PC3 cells resulted in larger lesions in tumor-bearing mice, which was blocked by inhibition of the β-catenin, a canonical WNT signaling pathway. Moreover, fibroblast-derived WNT16B attenuated mitoxantrone-induced cytotoxicity and apoptosis both in vitro and in vivo via β-catenin or NF-kB signaling [166].

Metabolic reprogramming has also been implicated in PCa chemoresistance. A shift towards oxidative phosphorylation (OXPHOS) has been captured in docetaxel-resistant PCa cells via downregulation of miR-205, whereas a shift towards glycolysis via miR205 re-expression restored chemosensitivity [167]. Patient-derived CAFs have been shown to drive this mitochondrial metabolic shift that has been associated with increased growth in PCa cells. Proteomic screening of conditioned media from primary patient-derived CAF and PCa co-cultures captured angiopoietin-like protein 4 (ANGPTL4) as a dominant CAF-secreted factor. ANGPTL4 binds IQGAP1 on the tumor cell surface, which induces mitochondrial biogenesis and boosts OXPHOS through the Raf-MEK-ERK-PGC1a signaling pathway. QGGP (Quercetin 3-O-(6′-galactopyranosyl)-β-D-galactopyranoside), an inhibitor of IQGAP1, restored docetaxel chemosensitivity of several PCa cell lines in the presence of primary CAFs [168]. An independent study by Pan et al. [169] identified cytosolic AKT-phosphorylated FOXO1 as another regulator of the IQGAP1/ERK axis. In preclinical solid tumor models including PCa cells, they demonstrated that AKT-phosphorylated FOXO1 inhibited ERK activation and chemoresistance by preventing FOXO1 nuclear translocation and blocking MAPK signaling. Therefore, disruption of the IQGAP1/ERK interaction is a potentially exploitable mechanism for restoring PCa chemosensitivity to taxanes by effects on both CAF and tumor cells within the PCa TME [169].

Altogether, a growing body of literature suggests that, in addition to mechanical remodeling of the TME, CAFs may play a significant role in promoting PCa chemotherapy resistance through paracrine CAF-derived factors. However, the translational utility of CAF-targeted strategies to restore chemosensitivity in treatment-resistant PCa requires further investigation.

#### 4.1.3. CAF Contribution in Radiotherapy Resistance

Radiotherapy (RT) is a commonly used treatment in clinical oncology with more than half of cancer patients receiving radiation to induce curative or palliative cytoreduction [170]. Ionizing radiation therapy efficacy relies on selectively killing tumor cells while minimizing damage to healthy cells [171]. Beyond inducing tumor cell death, RT has also been shown to boost anti-tumor immune responses via increased cell trafficking and epitope spreading during release of tumor antigens. The utility of combinatory ionizing radiation has been assessed in the context of immunotherapy resistance and found to improve immunogenic cell death [172,173]. Early clinical studies have suggested that combining RT, both stereotactic ablative body radiotherapy and radioligand therapy, with immunotherapy can yield dose-dependent effects and may delay tumor progression in PCa [174,175]. The role of CAFs in RT resistance has been studied in several tumor models, though controversy remains surrounding the specific mechanisms.

Sun et al. [166] showed that DNA-damaging therapies, including ionizing radiation, activated NF-κB in prostate fibroblasts. Conditioned medium from irradiated prostate fibroblasts increased proliferation, motility, and invasion of PCa cells. Additionally, co-implantation of irradiated fibroblasts resulted in larger tumor volumes in tumor-bearing mice when compared to non-irradiated fibroblast controls, which was attenuated by WNTB16 silencing. Furthermore, inhibition of β-catenin-mediated transcription diminished the WNTB16B-induced proliferative effects of irradiated fibroblast media. Mechanistic studies revealed that the pro-tumorigenic effects of irradiated fibroblast media were mediated by the NF-kB nuclear translocation and signaling activity in response to DNA damage; NF-kB-binding motifs were identified in the WNT16B promoter [166].

Radiation has also been associated with changes in CAF gene expression profiles. A study investigating a mouse CAF cell line resistant to RT identified 2626 DEGs that were associated with RT resistance. Human homologues were identified, and functional enrichment analysis on these DEGs showed enriched GO terms for ECM and immune-related pathways, including ECM structural constituent and regulation of leukocyte migration. This study reported favorable prognosis correlated with high *KCTD4* and *ACPP* as well as low *THBS2*. Shorter metastasis-free survival correlated with high expression of *HOPX*, *TMEM132A*, and *ZNF467*. This signature was found to be an independent prognosis factor for PCa patients; *ACPP* and *KCTD14* had significantly decreased and *THBS2* had increased expression in BCR versus non-BCR patients. In MET patients, *HOPX* and *TMEM132A* were upregulated. Finally, the high-risk CAF signature correlated with immune checkpoint expression (*PDCD1*, *TIGIT*, *ICOSLG*, *HLA-DOA*) suggesting that CAF gene programs may influence RT outcomes in PCa via regulation of stromal and immune microenvironment mechanisms [176]. Radiotherapy remains a crucial therapeutic tool in clinical management of advanced PCa. Therefore, further investigation is needed to explore potentially exploitable mechanisms within the TME interactome to restore radiosensitivity in high-risk disease.

### 4.2. CAF Targeted Therapies

In the past decade, various strategies have been assessed to disrupt tumor-promoting CAF interactions in both preclinical studies and clinical trials (Table 1 and Table 2). These strategies include depletion of CAFs, partial deletion of tumor-promoting CAF subsets, functional repolarization of CAFs, resetting CAF–TME interactions, blocking CAF differentiation, and blocking proteins that are significantly overexpressed by CAFs.

FAP has emerged as one of the most widely studied CAF-related targets, as it is overexpressed by CAFs and is not expressed in fibroblasts in normal tissues. Quantitative analysis of FAP expression in prostatectomy tissue found a strong association in “FAP area fractions” with high-risk forms of PCa, such as non-organ confined tumors and cribriform carcinoma morphology [194]. Enrichment of FAP^+^ stromal cell fraction particularly in MRI-visible lesions associated with PTEN status and predicted elevated risk for BCR. In this study, which included a total of 600+ prostatectomy cases from two independent cohorts, FAP overexpression was proposed as a strong negative prognostic biomarker [195]. Since FAP is abundant in tumor-associated fibroblasts and is largely restricted to the stroma, while normal prostate tissue regions (not affected by inflammation or atrophy) show virtually no expression [194], this biomarker is considered an enticing target for therapeutic development. Several FAP-targeted agents have shown promise in limiting tumor growth in preclinical murine models, including FAP^+^-CAR T cell-mediated targeting of desmoplastic pancreatic tumors [90] that are associated with reversal of immune exclusion and sensitized tumors to anti-PD-1 therapy. However, translational development of FAP-targeted CAR-T or T-cell therapies in PCa is limited and FAP-specific cellular immunotherapy in PCa remains a conceptual possibility for the time being.

Antibody–drug conjugates (ADC) provide an alternative method to achieving biomarker-specific delivery of cytotoxic agents while limiting off-target systemic exposure. Currently, there are several tumor cell-targeted ADCs in clinical assessment in PCa cohorts, such as TROP2-targeting Sacituzumab Govitecan (NCT03725761) and PSMA-directed ARX517 (NCT04662580), to treat mCRPC alongside prior PSMA-ADC clinical datasets [196,197,198]. Trophoblast cell-surface antigen 2 (TROP2) is a cell surface glycoprotein with high expression in CRPC and NEPC that functions as a driver of metastatic disease and a biomarker for biochemical recurrence and prostate-specific membrane antigen (PSMA) is a critical prognostic and diagnostic biomarker in PCa [199,200,201].

CAF-targeted ADCs are currently in development. A first-in-kind Phase I dose escalation study to assess efficacy of FAP-targeted ADC, OMTX705, with a tubulysin payload, in combination of anti-PD1 immunotherapy is currently in progress for patients with advanced solid tumors (NCT05547321). The preclinical efficacy of a novel FAP-specific (huB12) ADC with Monomethyl auristatin E (MMAE) payload has been recently investigated for targeting PCa-associated CAFs [180]. FAP-specific uptake of huB12-MMAE by FAP-expressing target cells was shown both in vitro and in vivo. HuB12-MMAE showed significant increase in FAP-specific cytotoxicity in FAP^+^ CAF cultures and improved survival in tumor-bearing mice with FAP-overexpressing xenografts. Additionally, huB12-MMAE-targeting of CAFs induced elevated CAF-derived secretion of several inflammatory cytokines, including IL-6 and IL-8.

Instead of depletion, another FAP-targeted strategy may aim at CAF inactivation. Successful delivery of siRNA into CAFs has been reported with a nanodelivery system. In an orthotopic PCa model, FAP-α–antibody linked to cell-penetrating peptide-based nanocarrier of siRNA was shown to effectively silence CAF-derived CXCL12, inactivate prostate CAFs and disrupt their interactions with tumor and endothelial cells including suppression of angiogenesis, invasion and metastasis [179].

An alternative approach to FAP-directed tumor targeting is the use of isomeric FAP inhibitors. Talabostat (BXCL701) is an oral dipeptidyl peptidase inhibitor that targets FAP, DPP4, and DPP8/9. However, mechanistic studies indicate that BXCL701 activity is complex and not strictly FAP-dependent, since DPP8/9 inhibition may trigger caspase-1–dependent pyroptosis and innate immune activation in myeloid cells [202]. Therefore, clinical data points do not solely reflect the isolated inhibition of the CAF/FAP axis. In phase Ib/II studies (NCT03910660) in heavily pretreated mCRPC, patients including both treatment emergent and *de novo* SCNC phenotypes received combination of BXCL701 and pembrolizumab [203]. Although early results showed a low response rate, persistent partial response and longer stable disease have also been observed in this cohort with acceptable safety profiles. This suggests the potential utility of small molecule inhibition of FAP with combination immunotherapy for advanced, treatment-resistant mCRPC.

Radionuclide therapy is designed to deliver localized, target-specific radiation to induce cytotoxicity. FAPI is a novel family of small molecule FAP inhibitors and quinoline-based FAPI development started in 2018 and has been used as a highly efficient vector in the context of radioligands with multiple formulations currently in clinical trials [204,205]. ^177^Lu-FAPI is a β-emitter which, due to its average range of 0.67 mm, maximum 1–2mm, also affects neighboring tumor cells and endothelium through a “cross-fire” effect [206,207]. In 2023, Laudicella et al. published a systematic review on FAPI-based theranostics in PCa. The review highlighted that FAPI data was largely limited to imaging applications in PCa and preliminary data on FAPI radioligand therapy was only reported in a few small cohorts [208]. Although PCa-specific subgroup analyses have not yet been published, an important context is provided by large FAP basket trials and other ^177^Lu-FAP developments. The LuMIERE phase I/II trial (NCT04939610) is a multicenter, open-label, non-randomized trial that has been evaluating ^177^Lu-FAP-2286 radioligand therapy in patients with advanced FAP-expressing solid tumors. An early report found the treatment to be well tolerated, and early results suggested preliminary evidence of antitumor activity [181].

A pilot study assessing ^177^Lu-FAPI-46 in patients with advanced tumors enrolled two patients with PCa. One of the two patients, who had bone metastatic PCa, received one cycle of treatment and showed stable disease [182]. ^90^Y-FAPI-46 therapy was assessed in a cohort of 21 patients with FAP-expressing metastatic solid tumors, including one patient with PCa, and was discontinued after the first cycle due to low response rate. Another pilot study assessing safety and efficacy of ^213^Bi-FAPI-46 RPT, an alpha-emitter with favorable pharmacokinetics, enrolled six patients with advanced solid tumors including one patient with PSMA-negative metastatic PCa who showed partial response with a 12% increase in post-treatment PSA [184]. Though several formulations of FAP-targeted radioligands are currently under preclinical and translational evaluation, as highlighted above, the clinical utility of FAPI therapeutics is yet to be fully developed.

PCa tumors are reportedly poor responders to immune checkpoint blockade (ICBs) [209], partly due to the reactive stroma enforcing an immune-excluded TME. CAF-derived TGFβ and ECM remodeling both physically and biochemically block CD8^+^ T-cell trafficking, while myeloid-polarizing signals further reduce antitumor immunity, thereby limiting ICB efficacy. Targeting TGFβ signaling and other paracrine pathways provide a novel approach to disrupt TME interactions that promote tumor growth. TGFβ inhibitors have appeared in several early-phase PCa trials but have yet to yield clinical breakthrough. SRK-181, a monoclonal antibody which binds to latent TGFβ to inhibit TGFβ activation, has been under evaluation both as a monotherapy and in combination with pembrolizumab for patients with solid tumor cancers in a Phase I clinical trial (NCT04291079, DRAGON trial). Thus far, of the 10 patients undergoing combination therapy, one patient had RECIST partial response (PR) and two patients had best response of stable disease (SD). Of the 15 patients receiving SRK-181 as a monotherapy, six patients, including one with PCa, had best response of SD [210]. Although conceptually appealing, targeting the CAF/TGFβ-rich stromal environment is not yet clinically mature.

In addition to TGFβ, IL-6 inhibition in the prostate appears to be an attractive concept, although early trials to block IL-6 as monotherapy did not yield clinical benefit. In a 2010 Phase II clinical trial (NCT00433446) for chemotherapy-exposed CRPC patients, Siltuximab, an anti-IL-6 antibody, did not show significant clinical benefit. Despite evidence of Siltuximab-mediated IL-6 inhibition, elevated baseline IL-6 levels were associated with poor prognosis [185]. IL-6 targeting has undergone several phase II trials with no clinical benefit, suggesting that although the CAF-IL-6 axis is biologically important in the context of chemoresistance and immunomodulation, its neutralization alone is not sufficient for breakthrough treatment of “cold” mCRPC tumors.

LY2510924 is a peptide antagonist that blocks CXCL12 from binding to CXCR4. A Phase I study investigating advanced solid tumors included patients with PCa. The drug was found to be safe, with LY2510924 demonstrating CD34^+^ cell mobilization, but no RECIST objective responses were observed [188].

Although targeting CAF-derived paracrine factors as a monotherapy has not been clinically successful, there is preclinical evidence showing that combinatory strategies may enhance tumor killing. The above-mentioned DRAGON trial includes an arm that assesses combination of TGFβ and anti-PDL1 checkpoint blockade in patients with solid tumors with promising early results. CXCR4 blocking was shown to enhance docetaxel cytotoxicity in a preclinical bone metastatic model of PCa [163]. Additionally, in a CRPC model, blocking the NF-KB pathway was sufficient to restore docetaxel chemosensitivity in PCa cell lines [190]. Inhibition of IL-6 under androgen deprivation was also associated with delayed progression of androgen-dependent PCa targets to androgen independence in vitro and in vivo [187]. However, an early clinical trial in 2012 evaluating the combination of the anti-IL-6 antibody Siltuximab with chemotherapy mitoxantrone/prednisone in docetaxel-exposed patients with mCRPC was terminated due to lack of improved outcomes [186].

IL-6 is a key immune regulating factor in the TME; therefore, anti-IL-6 therapy may favorably complement immune therapy strategies. An open-label Phase II neoadjuvant trial (NCT03821246) is currently recruiting patients to evaluate one cycle of atezolizumab (PD-L1 inhibitor) with or without IL-6R antibody tocilizumab/etrumadenant (adenosine receptor antagonist) in localized PCa. The primary goal of the trial is to investigate changes in the prostate tissue T-cell infiltrate at baseline and after therapy. Combination therapy with CXCR4 antagonists, ICB pembrolizumab and chemotherapy is currently being investigated in pancreatic ductal adenocarcinoma in a Phase IIa clinical trial and showed some evidence of an enhanced anti-tumor immune infiltrate (NCT02826486) [189].

Early assessment of clinical trials evaluating VEGFR, PDGFR and FGFR targeting key pathways in CAF-related regulation of TME organization and functions have been ongoing but have yet to achieve a translational breakthrough. A recent Phase II clinical trial assessing efficacy of Dovitinib, a pan-inhibitor of VEGFR, PDGFR and FGFR in 44 patients with PCa (NCT01741116) reported that 19 out of 44 patients showed some clinical benefit with longer PFS in the chemotherapy-naïve subcohort [191]. Biomarker analysis captured longer PFS in patients with higher baseline VEGFR2 plasma levels beyond the median. Early results from three clinical trials (NCT03654547, NCT04742959, NCT05253053) assessing Tinengotinib monotherapy, a multi-kinase inhibitor whose targets include VEGFRs and FFGRs, in 30 patients with treatment refractory mCRPC showed promising clinical response [211]. Additional PCa CAF-targeted therapies not discussed in detail in this review are listed in Table 2 [177,178,183,192,193].

Overall, while these therapies show promise, clinical studies to assess the translational utility of CAF-targeting strategies including CAF deletion or CAF reprogramming remain limited and need to be further developed in PCa.

## 5. Organ-on-a-Chip Models of CAFs

Over the past few decades, our understanding of PCa tumor biology has significantly advanced due in part to the diverse array of research models developed to study complex tumor biology. The most utilized models for investigating tumorigenesis include 2D cell cultures, 3D organoids, xenografts, allografts, transgenic mouse models, and organ-on-a-chip models. However, each of these models have distinct applications, advantages, and limitations. For the development of effective PCa therapies, it is crucial to mimic the complexity of interactions between tumor cells and the heterocellular TME networks recognizing the important role of stroma in PCa pathogenesis. However, investigating the dynamic interactions between tumors and stroma poses a formidable challenge when using traditional cell culture methods. While in vivo murine model systems may simulate some complex TME interactions, they lack key components that are specific to human physiology limit discovery. The development of more translationally accurate model systems is essential to facilitate development of novel strategies to treat PCa, particularly through the molecular pathways that drive resistance to androgen pathway-targeted therapies and progression to metastatic disease. These processes have been shown to be driven, in part, by the TME. PCa organ-on-a-chip (OOC) models could be used to assess many different elements of the TME, such as nutrient gradient, oxidative stress, and vessel development, that has been achieved by other non-cancer related models [212,213,214] and some progress has been made into these topics in other cancers such as breast cancers [215]. Current research efforts have concentrated on finding predictive biomarkers and developing combination therapies to improve patient outcomes. Combining 2D and 3D cultures to simulate more complex interactions has led to the rise in OOC models that allow integrated modeling of heterocellular networks in the context of native tissue-like spatial configurations. OOC models effectively mimic physiological characteristics in the microenvironment for many different organs, making them valuable for investigating complex biology in multicellular niches, such as the TME.

**Figure 3:**
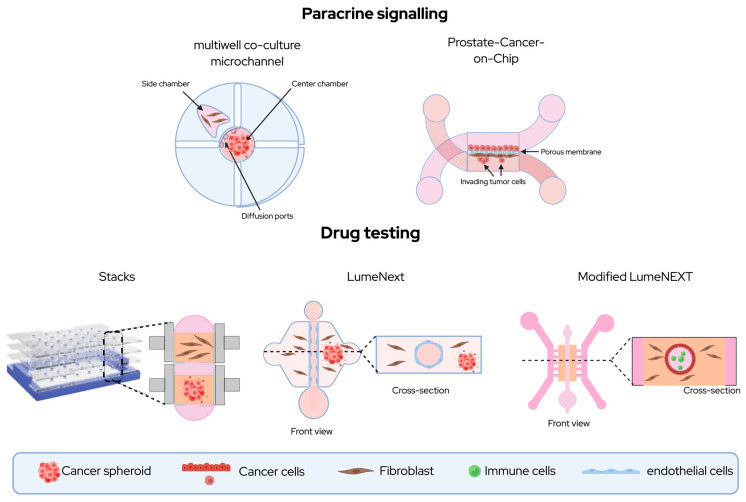
**Organ-on-Chip model systems established to study the prostate cancer tumor microenvironment.** The “multiwell co-culture microchannel model” and the Prostate-on-a-Chip facilitate the interrogation of paracrine signaling dynamics within the PCa TME. In the “multiwell co-culture microchannel” design, a central chamber is connected to radially oriented microchannels that lead to an outlet track, while adjacent side chambers communicate with the central chamber through diffusion ports to enable controlled molecular exchange. In the Prostate-Cancer-on-Chip device, prostate fibroblasts and epithelial cells are seeded on opposite sides of a semipermeable membrane, allowing paracrine interactions while preventing direct physical contact. The STACKs and LumeNEXT systems are microfluidic platforms that allow reconfigurable spatiotemporal investigation of multi-cellular co-culture systems, such as the PCa TME. Created in Biorender.com.

Several microfluidic models of TME were developed to assess novel strategies including rapid drug cultures or stimulating immunity [131,216,217] in the context of stromal–tumor crosstalk [218]. For example, the “multi-well co-culture microchannel model” has been developed as a suitable platform to study interactions in complex multi-cellular microenvironments, such as the PCa TME. Just in the last few years, several review articles have summarized the application, advantages, and future directions of microfluidic or microphysiological model systems (MPS) [219,220,221,222,223,224,225]. In this section, we will provide examples of OOC models that explore and investigate specific aspects of the role of tumor stroma in PCa (Figure 3).

One of the first OOC models developed to specifically model the human prostate tissue microenvironment was introduced by Jiang et al. and referred to as the Prostate-on-a-Chip (PoC). The PoC platform was designed to allow investigation into how prostate stroma induces and maintains epithelial differentiation. This design featured co-culture of stromal and epithelial prostate cells to simulate paracrine communication and effectively demonstrated how basal cells differentiate into luminal secretory cells through an AR-mediated mechanism [226]. Subsequently, Jiang et al. developed another model to simulate the PCa TME. The Prostate-Cancer-on-Chip (PCoC) incorporated co-culture of human PCa cells and stromal fibroblasts in two channels separated by a porous membrane, allowing media flow through and diffusion of secreted signaling factors [227]. This model was utilized to investigate the conversion of prostate fibroblasts to CAFs driven by heterocellular interactions and the study team reported detection of multiple CAF biomarkers, specifically α-SMA and COL1A1, in stromal fibroblasts in the context of tumor cells. The study revealed that the level of stromal conversion increased along the flow direction, aligning with diffusion patterns and simulations of solute concentration gradients. Additionally, the platform was utilized to examine tumor invasion into the stroma in the context of the metastatic cascade. The study team reported that both tumor cells and CAFs infiltrated the neighboring channel. Overall, the PCoC platform is a promising tool to analyze the interaction between tumor cells and the surrounding stroma to clarify biological mechanisms in the background.

In 2019, the STACKs microfluidic platform was developed for multi-culture investigations with reconfigurable, stackable layers that allow investigation of spatiotemporal interactions between various cell subsets including PCa tumor–stromal networks [228]. In a recent study, the STACKs platform was utilized to assess the efficacy of a novel FAP-specific ADC in targeting of PC-associated CAFs in the context of an MMAE payload, a potent antineoplastic agent that inhibits tubulin polymerization. This STACKs model allowed for rapid in vitro assessment of FAP-MMAE efficacy in disrupting the interactions between components of the tumor microenvironment (TME), and endpoints assessable included enhancement of tumor cell death and immune infiltration in the context of CAF–tumor co-cultures [180]. Multiplexed analysis of mRNA and protein expression in STACKs further revealed that treatment with FAP-MMAE induced secretion of CAF-derived pro-inflammatory cytokines, as well as overexpression of pro-inflammatory genes in the targeted tumor cells, providing rationale for further assessment of translational utility of this novel CAF-targeted ADC.

Spatial organization of the TME has relevant implications in tumor biology including factors that affect ECM structures, intercellular interactions, and cell trafficking. However, most in vitro systems have limitations in modeling these complex configurations. Therefore, creating MPS that allow the recreation of translationally relevant tissue architectures is an important element of accurate simulation of TME interactions. Certain types of tumors, including breast, kidney, and prostate cancers, typically originate from tubular structures called lumens. Lumens are also found in blood vessels and the lymphatic system with important effects on the bi-directional TME trafficking events such as tumor-immune infiltration and the early metastatic cascade. Therefore, to investigate PCa tissue biology, lumen-based MPS, provide additional value by recreating spatial organization. The LumeNEXT platform is a closed microfluidic system that allows the formation of a microfluidic chamber with integrated lumens to allow modeling vascularized PCa TME. The model has been established for organotypic modeling, studying cell interactions, and drug screening. The device was successfully validated as a platform to study interactions between cancer cells and stromal cells including CAF-driven tumor cell trafficking [229].

The LumeNEXT system has been established to mimic prostate ducts across various stages of PCa in the context of PCa TME elements, such as infiltrating immune cells, and CAFs [131]. Furthermore, the LumeNEXT platform has been utilized for investigating gene expression profiles as well as the metabolic and paracrine signaling landscapes of the PCa TME for various stages of the disease in the context of CAFs and other stromal elements. In a recent report, the LumeNEXT device was adapted to serve as a patient-specific platform to mimic the PCa-associated metastatic bone TME. The LumeNEXT-based PCa bone chip allows assessment of treatment response patterns in the context of stromal effects across fibroblasts, osteoclasts, osteoblasts, and adipocytes by multi-parameter confocal microscopy, RT-qPCR and multiparameter protein bead array. Additionally, protocols have been established for scRNA-seq transcriptomic profiling of these chips [230]. The ability to accurately model primary and metastatic sites of PCa opens many possibilities for understanding induction of metastatic cascade, preferential localization of tumor cells to metastatic microenvironments, and the intravasation through tissue barriers, such as blood or lymphatic vessels for colonization of distant organs. The “modified LumeNEXT” system is adapted from the original LumeNEXT design, in which two PDMS layers are assembled around a removable rod to generate a central collagen-based lumen, which can be subsequently lined with prostate epithelial cells to model the prostate ductal architecture. Two flanking media channels are integrated on either side of the main chamber. These channels allow continuous exchange of culture media while preserving the integrity of the lumen environment, where additional PCa TME cells, such as immune cells, can be introduced to study more complex TIME interactions.

In recent years, several OOC models have been developed to study PCa that provide promising value to enhance our understanding of how the microenvironment impacts tumor progression and treatment response patterns. Though the physiological complexity of in vivo animal models may be difficult to capture in OOC models, multi-organ vascularized OOC models are a rapidly growing area of OOC research. Broadly, these humanizable, multi-analyte OOC systems open new capabilities for precision disease modeling and expedite preclinical development [231,232,233,234]. TME elements play a crucial role in shaping therapeutic efficiency and OOC platforms may bridge a gap by providing translationally more relevant, fully humanized microenvironments including patient matched chips in “n of 1 trials” to uncover molecular mechanisms behind resistance.

## 6. Conclusions

In PCa, CAFs represent the most prevalent stromal cell type with critical roles in tumor progression processes, including cell growth, invasion, and resistance. Although CAFs have been extensively studied as pivotal contributors to cancer development and present an appealing therapeutic target, clinical development of CAF-targeted therapies has yielded limited success thus far. Furthermore, there is a pressing need for the development of novel combinatorial therapies that adequately consider TME response to anti-androgens, chemotherapy, and radiotherapy and effectively address the role of TME in the development of treatment-resistant PCa. However, the inherent molecular heterogeneity of CAFs, coupled with the lack of exclusive biomarkers, maintains significant challenges to achieving these aims. Consequently, understanding the diverse functionalities of CAFs in the context of native TME networks is imperative to identifying novel biomarkers and exploitable driver mechanisms for CAF-targeted therapeutic gain. Additionally, focused studies on expanded and longitudinal clinical cohorts will be instrumental to establishing key common characteristics of the translationally relevant CAF subsets in human PCa. Since animal and conventional cell culture models are limited in recapitulating the dynamic TME in the context of human tissue physiology, OOC models are becoming a relevant tool to provide fully humanized platforms to advance PCa research. OOC models are poised to play a significant role in this endeavor and provide new capabilities to revolutionize many aspects of the translational workflow. This includes expediting preclinical assessment of novel CAF-targeted therapeutics and personalized medicine strategies in the context of the TME with integrated vascular, neuronal and immune networks, and with the potential of creating fully patient-matched TMEs. Integrated multi-analyte capabilities of OOC platforms may significantly shorten preclinical efficacy testing of novel agents including radiopharmaceuticals, nanodelivery systems, novel immunotherapiesand combinatory approaches to develop more effective treatment modalities to enhance care for patients with high-risk, treatment-resistant prostate cancer.

## Figures and Tables

**Table 1:** CAF FAP-based targeted clinical trials and targeted therapies in development for PCa.

Target/Mechanism	Drug	Phase	Patient Population	Clinical Trial Number	Publication	Status
FAP	Combination of BXCL701 Pembrolizumab, anti-PD1	Phase Ib/II	mCRPC with small cell NEPC or Adenocarcinoma	NCT03910660		Ongoing
	Combination of FAP cleavable peptide and Doxorubicin (CAP-NP)	Preclinical	PC-3 Xenografts		[177]	
	HA@DSP-pep-DSP (Dox- loaded poly-amidoamine) nanoparticles	Preclinical	PC-3 Xenografts		[178]	
	FAP antibody+ CPP based nanoparticles loaded with siRNA and CXCL12 ligands	Preclinical	PC-3 Xenografts (Orthotopic)		[179]	
FAP (CAF depletion) FAP^+^-CAR T cell	FAP^+^-CAR T	Preclinical	Desmoplastic pancreatic tumors (murine models)		[90]	Preclinical
FAP (CAF-targeted ADC)	OMTX705 (tubulysin payload) + anti-PD1	Phase I (dose escalation)	Advanced solid tumors	NCT05547321		In progress
FAP (CAF-targeted ADC)	huB12-MMAE (MMAE payload)	Preclinical	PCa-associated CAF targeting; xenograft models		[180]	Preclinical
FAP-α targeting siRNA delivery/CAF inactivation (CXCL12 silencing)	FAP-α Ab–linked CPP-nanocarrier (siRNA anti-CXCL12)	Preclinical	Orthotopic PCa model		[179]	Preclinical
FAP/DPP inhibition (complex MoA; incl. DPP8/9 → pyroptosis/innate activation)	Talabostat (BXCL701) + Pembrolizumab	Phase Ib/II	Heavily pretreated mCRPC incl. treatment-emergent & *de novo* SCNC phenotypes	NCT03910660		Ongoing/early results reported
FAP-targeted radioligand therapy	^177^Lu-FAP-2286	Phase I/II	Advanced FAP+ solid tumors (basket)	NCT04939610	[181]	Ongoing; early report published
FAP inhibitor radioligand (FAPI-46)	^177^Lu-FAPI-46	Pilot (small cohort)	Advanced tumors; included 2 PCa patients		[182]	Reported (pilot)
FAP inhibitor radioligand (FAPI-46)	^90^Y-FAPI-46	Cohort/pilot	21 metastatic FAP-expressing solid tumors; included 1 PCa patient		[183]	Discontinued after 1st cycle (low response rate)
FAP inhibitor radioligand (FAPI-46, alpha-emitter)	^213^Bi-FAPI-46	Pilot (small cohort)	Advanced solid tumors; included 1 PSMA- metastatic PCa		[184]	Reported (pilot)

**Table 2:** Other CAF-targeted clinical trials and targeted therapies in development for PCa.

Target/Mechanism	Drug	Phase	Patient Population	Clinical Trial Number	Publication	Status
IL-6 neutralization	Siltuximab	Phase II	Chemotherapy-exposed CRPC	NCT00433446	[185]	Completed (no significant benefit)
IL-6 pathway inhibition to “warm up” TME (neoadjuvant ICB context)	Atezolizumab ± Tocilizumab (IL-6R Ab)/Etrumadenant (adenosine receptor antagonist)	Phase II (open-label, neoadjuvant)	Localized PCa prior to radical prostatectomy	NCT03821246		Recruiting
IL-6 blockade + chemotherapy	Siltuximab + Mitoxantrone/Prednisone	early clinical trial in 2012	mCRPC prior docetaxel		[186]	Terminated early (no improved outcomes)
IL-6 inhibition under ADT (progression delay)	Atorvastatin + Celecoxib	Preclinical	Androgen-dependent PCa → androgen independence (*in vitro*/*in vivo*)		[187]	Preclinical
TGFβ pathway (CAF-derived TGFβ;stromal immune exclusion)–latent TGFβ activation blockade	SRK-181 (anti-latent TGFβ) ± Pembrolizumab	Phase I	Solid tumors; combo & monotherapy cohorts (1 PCa with SD)	NCT04291079		Ongoing; early results reported
CXCL12/CXCR4 axis receptor blockade	LY2510924 (CXCR4 peptide antagonist)	Phase I	Advanced solid tumors including PCa		[188]	Completed/reported (no RECIST response)
CXCR4 blockade + chemo-therapy (preclinical synergy)	Balixafortide + Docetaxel	Preclinical	Bone metastatic PCa model		[163]	Preclinical
CXCR4 antagonist + ICB + chemo (stromal/immune-infiltrate context)	BL-8040 (CXCR4 antagonist) + Pembrolizumab + chemotherapy	Phase IIa	Pancreatic ductal adenocarcinoma	NCT02826486	[189]	Reported (evidence of enhanced immune infiltrate)
NF-κB pathway inhibition (chemosensitization; CAF-paracrine context)	NF-κB inhibition (p65 siRNA)	Preclinical	CRPC model; PCa cell lines (docetaxel re-sensitization)		[190]	Preclinical
FGFR1-3, VEGFR1-3, PDGFRα, PDGFRβ, Src, Lck, Lyn	Nintedanib or Afatinib	Phase II	Hormone-refractory PCa	NCT00706628		Completed
FGFR1-3, VEGFR1-3, PDGFRβ, FLT-3, c-KIT	Dovitinib	Phase II	CRPC patients with bone metastases	NCT00831792		Completed
FGFR1-3, VEGFR1-3, PDGFRβ, FLT-3, c-KIT	Dovitinib	Phase II	mCRPC, post Docetaxel resistance	NCT01741116	[191]	Completed (no results posted yet)
FGFR	Tinengotonib (Multi Kinase Inhibitor; AURORA, VEGFR, FGFR, JAK, CSF1R)	Phase I/II	Advanced Solid Tumors	NCT03654547, NCT04742959, NCT05253053		Completed
	Nab-Paclitaxel	Phase Ib/Phase I	Advanced Solid Tumors	NCT02048943		Withdrawn
GFRAL	NGM120 (Anti-GFRAL Ab)	Phase I/II	Advanced solid tumors, mCRPC	NCT04068896		Completed
VEGFR	Cabozantinib (Multiple Kinase Inhibitor; MET, VEGFR2, RET)	Phase I	Locally advanced or metastatic solid tumors, mCRPC	NCT03170960		Completed
	Atezolizumab (anti PD-L1 Ab)	Phase I				Completed
	PTK787 (VEGFR inhibitor)	Phase II	Non-Metastatic Androgen Independent Pca	NCT00134355		Completed
	Pazopanib (VEGFR inhibitor)	Phase II	High-Risk PCa	NCT01832259		Completed
	Axitinib (VEGFR inhibitor)	Phase II	High-Risk PCa	NCT01385059		Completed
	ESK981 (TIE2, VEGFR1-3 and FGFR1 inhibitor)	Phase II	mCRPC	NCT03456804		Completed
	Bevacizumab + anti VEGF mAb + Temsirolimus (mTOR inhibitor)	Phase I/II	Hormone-Resistant Metastatic Pca	NCT01083368	[192]	Completed
	Tivozanib (Tyrosine Kinase Inhibitor) + Atezolizumab	Phase I/II	Immunologically Cold Tumors	NCT05000294	[193]	Suspended for interim analysis
EphB2	sEphB4-HAS (EphrinB2 inhibitor) + Pembrolizimab	Phase II	PCa and Urothelial Carcinoma	NCT02717156		Ongoing

## Data Availability

No new data were created or analyzed in this study. Data sharing is not applicable to this article.

## Abbreviations

The following abbreviations are used in this manuscript:

CAF	Cancer-Associated Fibroblast
TME	Tumor Microenvironment
PCa	Prostate Cancer
PSA	Prostate Specific Antigen
ADT	Androgen Deprivation Therapy
ARPI	Androgen Receptor Pathway Inhibitor
CRPC	Castrate Resistant Prostate Cancer
AR	Androgen Receptor
ECM	Extracellular Matrix
MSC	Mesenchymal Stem Cell
NF	Normal Fibroblast
FAP	Fibroblast Activation Protein
myCAF	Myofibroblastic CAF
apCAF	Antigen presenting CAF
iCAF	Inflammatory CAF
MDSC	Myeloid Derived Suppressor Cells
T_reg_	Regulatory T cell
NEPC	Neuroendocrine Prostate Cancer
proCAF	Progenitor CAF
matCAF	Matrix Producing CAF
TAM	Tumor Associated Macrophage
ICB	Immune Checkpoint Blockade
MMAE	Monomethyl auristatin E
